# Factors associated with willingness to take extended release naltrexone among injection drug users

**DOI:** 10.1186/s13722-015-0034-5

**Published:** 2015-05-03

**Authors:** Keith Ahamad, MJ Milloy, Paul Nguyen, Sasha Uhlmann, Cheyenne Johnson, Todd P Korthuis, Thomas Kerr, Evan Wood

**Affiliations:** British Columbia Centre for Excellence in HIV/AIDS, St. Paul’s Hospital, 1081 Burrard Street, Vancouver, BC V6Z 1Y6 Canada; Department of Family Practice, University of British Columbia, 5950 University Boulevard Street, Vancouver, BC V6T 1Z3 Canada; Department of Medicine, University of British Columbia, 10th Floor 2775 Laurel Street, Vancouver, BC V5Z 1M9 Canada; Department of Medicine, Oregon Health and Science University, 3181 SW Sam Jackson Park Rd, Portland, OR 97239 USA; Department of Public Health-Preventive Medicine, Oregon Health and Science University, 3181 SW Sam Jackson Park Rd, Portland, OR 97239 USA; Division of Epidemiology and Population Health, BC Centre for Excellence in HIV/AIDS, 608-1081 Burrard Street, Vancouver, BC V6Z 1Y6 Canada

**Keywords:** Addiction, Willingness to take, Opioid antagonist

## Abstract

**Background:**

Although opioid-agonist therapy with methadone or buprenorphine/naloxone is currently the mainstay of medical treatment for opioid use disorder, these medications often are not well accepted or tolerated by patients. Recently, extended release naltrexone (XR-NTX), an opioid antagonist, has been advanced as an alternative treatment. The willingness of opioid-addicted patients to take XR-NTX has not been well described.

**Methods:**

Opioid-using persons enrolled in a community-recruited cohort in Vancouver, Canada, were asked whether or not they would be willing to take XR-NTX. Logistic regression was used to independently identify factors associated with willingness to take the medication.

**Results:**

Among the 657 participants surveyed between June 1, 2013, and November 30, 2013, 342 (52.1%) were willing to take XR-NTX. One factor positively associated with willingness was daily heroin injection (adjusted odds ratio [AOR] = 1.53; 95% confidence interval [CI] = 1.02–2.31), whereas Caucasian ethnicity was negatively associated (AOR = 0.59; 95% CI = 0.43–0.82). Satisfaction with agonist therapy (13.4%) and unwillingness to stop opioids being used for pain (26.9%) were the most common reasons for being unwilling to take XR-NTX.

**Conclusions:**

A high level of willingness to take XR-NTX was observed in this setting. Interestingly, daily injection heroin use was positively associated with willingness, whereas Caucasian participants were less willing to take XR-NTX. Although explanations for unwillingness were described in this study, further research is needed to investigate real-world acceptability of XR-NTX as an additional option for the treatment of opioid use disorder.

## Background

Opioid use disorder remains a major public health concern worldwide. Globally, opioids top the list of illicit drugs that cause substantial morbidity and mortality [1]. In the United States alone, over 5 million people abused prescription pain relievers in 2010 [2]. Nonmedical users of prescription opioids have also been shown to be almost 8 times more likely to use heroin and over 4 times more likely to use intravenously [3].

While the health and social consequences of opioid use disorder are well documented, past studies have clearly demonstrated significant reductions in these consequences through medically assisted treatment with methadone or buprenorphine, which are the gold standards for medical management of opioid use disorder [4-7]. In terms of psychosocial treatments, past studies have generally shown that without opioid-agonist medications like methadone or buprenorphine, the vast majority of patients will be treatment-refractory to psychosocial and nonpharmacological interventions [4,8].

Unfortunately, due to the real or perceived side effects or programmatic characteristics, treatment with methadone and buprenorphine does not attract all users into treatment. Commonly reported barriers to program entry include limited treatment availability, the high threshold of treatment programs (i.e., safe dose titration timelines), and requirements that opioid-agonist medications must be provided via daily, witnessed ingestion [9]. Furthermore, while they vary from setting to setting, retention rates with opioid-agonist treatments are only approximately 60 percent, and many patients on methadone or buprenorphine should remain on this medication indefinitely [10-12]. Oral naltrexone, an opioid-receptor antagonist, has been proposed as an alternative for patients. Naltrexone has several advantages in that it has few drug–drug interactions, no physical dependence, and a favorable side-effect profile [13]. Unfortunately, due to the requirement for daily oral dosing, medication adherence has been the major barrier to its efficacy [14].

Recently, naltrexone for extended-release injectable suspension (XR-NTX), which is administered via intramuscular injection every 4 weeks, has been advanced as an alternative treatment [15,16]. Placebo-controlled trials have demonstrated its efficacy in retaining patients in treatment, increasing abstinence, and decreasing opioid cravings [15-17]. However, it has recently been noted that the willingness of opioid-addicted patients to take XR-NTX has not been well described [18]. Since little is known about the willingness of opioid-addicted patients to take an extended-release, opioid-antagonist medication (or about factors that may predict willingness), we undertook this study to examine the willingness to use XR-NTX among opioid-addicted patients participating in a cohort study in Vancouver, Canada.

## Methods

Data for this study were derived from the Vancouver Injection Drug Users Study (VIDUS), an open, prospective cohort of HIV-seronegative individuals who inject drugs, and the AIDS Care Cohort to Evaluate Access to Survival Services (ACCESS), an open, prospective cohort of HIV-seropositive individuals who use illicit drugs in Vancouver, Canada. Detailed methodology has previously been described [19,20]. Participants were eligible for the study if they were 18 years or older, used illicit drugs other than cannabis within the past month, resided in the Greater Vancouver region, and provided informed consent. Participants were recruited through extensive street-based outreach methods and snowball sampling, beginning in May 1996. At baseline and every 6 months thereafter, participants completed an interviewer-administered questionnaire that elicited information regarding sociodemographic characteristics, drug use, HIV risk behaviors, and treatment utilization, and underwent an examination by a nurse. Participants received a $20 CAD stipend for each visit. VIDUS and ACCESS study recruitment and follow-up procedures were essentially identical, with the exception of questions specific to HIV infection, to enable merged analyses. Both the VIDUS and ACCESS studies were ethically approved by the Research Ethics Board of Providence Health Care/University of British Columbia.

For the primary analysis, we restricted the study sample to those who reported any use of opioids or who enrolled in methadone maintenance therapy in the past 6 months, and assessed whether participants were willing to take XR-NTX for opioid addiction treatment by adding questions to follow-up visits between June 1, 2013, and November 30, 2013. Specifically, participants were asked: “There is a new medication that can be given to people who have detoxed from heroin or other opioids, including methadone. It is given by intramuscular injection. It completely blocks the effects of opioids, including pain medications, for 30 days. If you are on methadone or using other opioid drugs, would you be interested in taking this medication if it becomes available in Canada?” Since XR-NTX was not available in Canada at the time these questions were utilized, staff were trained to answer questions about the medication’s effects, induction, and duration of action. Participants who answered “Yes” were compared to those who answered “No” on a variety of *a priori*-selected sociodemographic, behavioral, and drug use variables hypothesized to be associated with willingness to take XR-NTX.

These variables included: age (per year older); female gender (yes vs. no); ethnicity (Caucasian vs. other); daily heroin injection (yes vs. no); daily cocaine injection (yes vs. no); daily crack smoking (yes vs. no); homelessness (yes vs. no); involvement in sex work, defined as exchanging sex for money, gifts, food, shelter, clothes, drugs, or other commodities (yes vs. no); HIV seropositivity (yes vs. no); or participation in drug treatment, defined as alcohol and/or drug treatment other than methadone treatment (yes vs. no). All behavioral and drug use characteristics refer to the 6-month period prior to the interview. All variable definitions have been used extensively and were identical to earlier publications [21,22].

We used bivariate and multivariate logistic regression analyses to determine factors associated with the willingness to take XR-NTX. To identify the independent correlates of willingness to take XR-NTX, only variables that were associated with willingness at *p-*value *<* 0.10 in bivariate analyses were considered in the full multivariate model. Using the backwards-selection procedure, we constructed the final multivariate model with the best fit, as indicated by the lowest Akaike Information Criterion (AIC) value.

As a sub-analysis among participants who did not report any willingness to take XR-NTX, their reasons from the subsequent question were collated. Participants could select more than one response from the following reasons: “Not using opioids”; “happy with methadone/suboxone (buprenorphine/naloxone)”; “don’t feel that I could detox from opioid use or reduce my methadone to zero”; “feel I could not get off my pain med”; “don’t want to take a long-acting medication by injection”; and “other”. All statistical analyses were performed using SAS software version 9.3 (SAS, Cary, NC, USA). All statistical tests were two-sided, with alpha = 0.05.

## Results

Between June 1, 2013, and November 30, 2013, 657 opioid-using VIDUS and ACCESS participants were interviewed and included in the present analysis. Among these individuals, median age was 48 (inter-quartile range = 41–53); 249 (37.9%) were female; and 397 (60.4%) were Caucasian (Table 1).

**Table 1:** **Characteristics of study participants assessed for willingness to take an opioid antagonist (N = 657)**

**Characteristic**	**Willingness**		**Odds ratio (95% CI** ^**£**^ **)**	***p*** **value**
	**No = 315 (%)**	**Yes = 342 (%)**		
**Age [Median, (IQR** ^¥^ **)]**	49 (41–54)	46 (40–51)	0.98 (0.96–1.00)	0.015
**Gender**				
Male	209 (66.3)	199 (58.2)		
Female	106 (33.7)	143 (41.8)	1.42 (1.03–1.95)	0.032
**Caucasian ethnicity**				
No	102 (32.4)	158 (46.2)		
Yes	213 (67.6)	184 (53.8)	0.56 (0.41–0.77)	< 0.001
Daily heroin injection*				
No	268 (85.1)	270 (79.0)		
Yes	47 (14.9)	71 (20.8)	1.50 (1.00–2.25)	0.050
Daily cocaine injection*				
No	289 (91.7)	319 (93.3)		
Yes	26 (8.3)	23 (6.7)	0.80 (0.45–1.44)	0.457
Daily crack smoking*				
No	251 (79.7)	279 (81.6)		
Yes	64 (20.3)	63 (18.4)	0.89 (0.60–1.30)	0.538
Homelessness*				
No	273 (86.7)	295 (86.3)		
Yes	42 (13.3)	46 (13.5)	1.01 (0.65–1.59)	0.953
Sex work*				
No	293 (93.0)	312 (91.2)		
Yes	22 (7.0)	30 (8.8)	1.28 (0.72–2.27)	0.397
**HIV positive**				
No	189 (60.0)	198 (57.9)		
Yes	126 (40.0)	144 (42.1)	1.09 (0.80–1.49)	0.584
Participation in drug treatment**				
No	257 (81.6)	275 (80.4)		
Yes	57 (18.1)	64 (18.7)	1.05 (0.71–1.56)	0.812

NOTE: Percentages do not necessarily sum to 100% due to missing data or rounding error.

*Activities in last 6 months.

**Defined as drug and/or alcohol treatment other than a methadone program.

^¥^IQR = inter-quartile range.

^**£**^CI = confidence interval.

Of the 657 participants, 342 (52.1%) indicated a willingness to take XR-NTX. As shown in Table 1, the sociodemographic, behavioral, and drug use characteristics associated with a willingness to take XR-NTX in unadjusted analyses included: age, female gender, daily heroin injection, and Caucasian ethnicity (all *p* < 0.05). The results of the multivariate analysis are presented in Table 2. Factors independently associated with a willingness to take XR-NTX included: daily heroin injection (adjusted odds ratio [AOR] = 1.53; 95% confidence interval [CI] = 1.02–2.31); and Caucasian ethnicity (AOR = 0.59; 95% CI = 0.43–0.82]).

**Table 2:** **Multivariate analysis of factors associated with the willingness to take an opioid antagonist (N = 657)**

**Characteristic**	**Adjusted odds ratio**	**95% CI** ^**£**^	***p*** **value**
Daily heroin injection*			
Yes vs. No	1.53	1.02–2.31	0.043
**Female gender**			
Yes vs. No	1.29	0.93–1.79	0.133
**Caucasian ethnicity**			
Yes vs. No	0.59	0.43–0.82	0.002

^**£**^CI = confidence interval.

*Activities in last 6 months.

**Figure 1:**
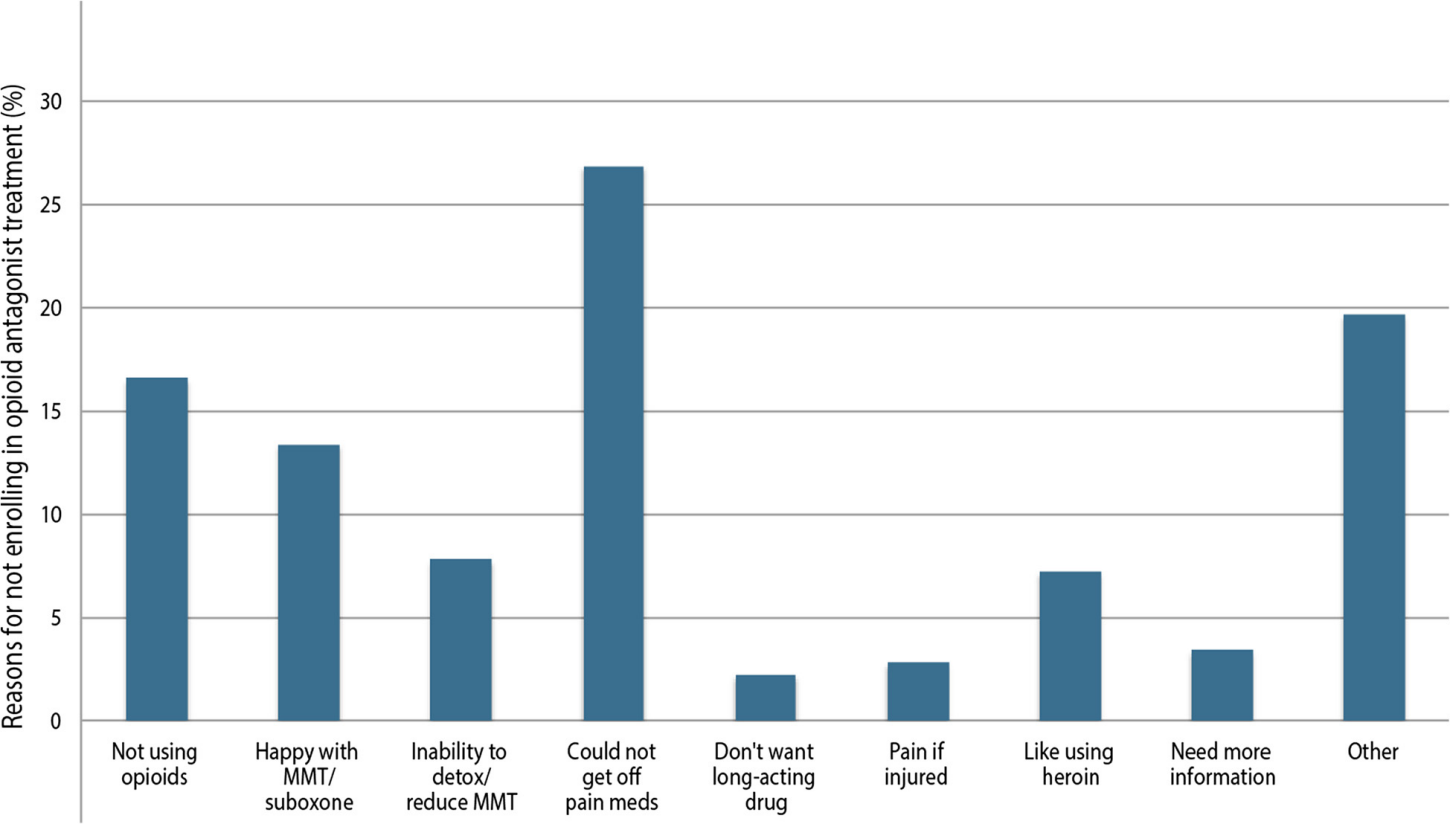
Reasons given by participants who indicated they would not be willing to take XR-NTX by percentage.

As shown in Figure 1, of the 315 (47.9%) participants who indicated that they would not be willing to take XR-NTX, 320 reasons were given for unwillingness and included: 53 (16.6%) reported that they were not actively taking opioids; 43 (13.4%) were happy with methadone/suboxone (buprenorphine/naloxone); 25 (7.8%) didn’t feel that they could detox from opioids; 86 (26.9%) didn’t feel that they could get off pain medications; 7 (2.2%) didn’t want to take a long-acting medication by injection; 9 (2.8%) reported concern regarding untreated pain if injured; 23 (7.2%) reported they liked using heroin; 11 (3.4%) reported needing more information; and 63 (19.7%) reported a variety of other reasons that were not consistent enough to be collapsed into categories. Data are available from the corresponding author.

## Discussion

In the present study, we found high rates of willingness to take XR-NTX among a community-recruited cohort of opioid drug users. We also found that daily heroin use was independently associated with a higher likelihood of willingness to take XR-NTX, whereas Caucasian participants were associated with a lower likelihood of willingness. Among the reasons reported for unwillingness to take XR-NTX: a) the perceived inability to stop opioid use for pain, and b) satisfaction with methadone/buprenorphine were the most frequently cited reasons.

This is the first study, to our knowledge, to assess patients’ interest in taking a long-acting opioid antagonist for the treatment of opioid addiction. Rather than patient-related factors, past studies have examined how individual and organizational variables can influence treatment program attitudes towards use of medications for the treatment of opioid addiction [23]. Fuller et al. (2005) surveyed outpatient substance abuse treatment centers and found that standalone substance abuse clinics were less likely to provide naltrexone [24]. Past studies have also examined adoption of naltrexone in the context of alcohol use disorder and found that only 15 percent of alcohol treatment clinicians prescribe naltrexone often [25]. In light of the dearth of information regarding patient profiles that may be appropriate for XR-NTX, Ling et al. outlined some of the questions regarding the role of XR-NTX in opioid dependence, including the fact that “no one seems to have figured out that perhaps we should ask our patients whether they would like to take [XR-NTX]” [18].

Our findings show a high degree of willingness to take XR-NTX, particularly among high-intensity heroin injectors. These results show there is a subset of opioid users who are willing to try alternatives to traditional opioid-agonist therapy. This may represent the fact that methadone has very high penetrance in Vancouver, and that those who have had a longstanding opportunity to engage in methadone treatment remain hesitant to take this medication for programmatic reasons (e.g., daily witness ingestion) and may be interested in an alternative [26]. Importantly, Kerr et al. showed in 2005 that opioid-injecting Aboriginal patients were less than half as likely to use methadone maintenance than non-Aboriginal persons [26]. In this regard, it is noteworthy that the majority of individuals in the non-Caucasian category in the present study were Aboriginal. While preliminary, our results suggest that there are likely reasons for the unwillingness to take opioid-agonist treatment that are more prevalent among Aboriginal persons, whereas this population may be more open to alternatives, including XR-NTX [26]. This is an issue that will require further study, potentially using qualitative research methods to explore this question and to examine willingness and unwillingness to take XR-NTX.

This study has limitations. As our study sample was generated through street-based recruitment methods, generalizing our findings to other populations of injection drug users requires caution. However, it is noteworthy that the cohort demographics are similar to other local and international studies of drug users [27-30]. Secondly, as our outcome of interest was willingness to take XR-NTX, actual rates of willingness and successful induction onto XR-NTX will need to be studied in clinical trials in real-world settings. In particular, all elements of the medications’ benefits and side-effect profile could not be fully described in the context of our study. In this regard, assessment of specific populations, including HIV-infected individuals, is the subject of ongoing investigation, as can be seen with the National Institute on Drug Abuse (NIDA) Clinical Trials Network (CTN) 055 CHOICES study [31]. Finally, socially desirable responding is a concern in studies of marginalized populations [32]. Although interviewers were trained to build trust and rapport with participants, and confidentiality was assured, it is possible we overestimated the percentage of individuals willing to participate as a result of this concern.

In summary, the present study found high rates of willingness to take XR-NTX in this setting. Interestingly, daily injection heroin use was independently and positively associated with willingness to take XR-NTX, while Caucasian participants were negatively associated with this choice, suggesting that sub-populations may benefit from this medication. Although patient-reported explanations for unwillingness were described in this study, including satisfaction with agonist therapy and concerns regarding stopping opioid-based pain medications, further research is needed to investigate real-world acceptability of XR-NTX as an additional option for the treatment of opioid use disorder.
